# Towards a Research Agenda for Promoting Responsible Research Practices

**DOI:** 10.1177/15562646211018916

**Published:** 2021-05-26

**Authors:** Joeri K. Tijdink, Serge P.J.M. Horbach, Michèle B. Nuijten, Gareth O’Neill

**Affiliations:** 1 Department of Ethics, Law and Humanities, 1209 Amsterdam UMC, Amsterdam, the Netherlands; 2 Department of Philosophy, 404761 Vrije Universiteit Amsterdam, Amsterdam, the Netherlands; 3 Danish Centre for Studies in Research and Research Policy, Aarhus University, Aarhus, Denmark; 4 Center for Science and Technology Studies, 168095 Leiden University, Leiden, the Netherlands; 5 Department of Methodology and Statistics, Tilburg School of Social and Behavioural Sciences, 120694 Tilburg University, Tilburg, the Netherlands; 6 Technopolis Group, Brussels, Belgium; 7 Leiden University Centre for Linguistics, Leiden University, Leiden, the Netherlands

**Keywords:** responsible research practices, research integrity, research agenda, research ethics, authorship, conflicts of interest/bias, educational research, questionable research practices

## Abstract

This opinion piece aims to inform future research funding programs on responsible research practices (RRP) based on three specific objectives: (1) to give a sketch of the current international discussion on responsible research practices (RRPs); (2) to give an overview of current initiatives and already obtained results regarding RRP; and (3) to give an overview of potential future needs for research on RRP. In this opinion piece, we have used seven iterative methodological steps (including literature review, ranking, and sorting exercises) to create the proposed research agenda. We identified six main themes that we believe need attention in future research: (1) responsible evaluation of research and researchers, (2) the influence of open science and transparency on RRP, (3) research on responsible mentoring, supervision, and role modeling, (4) the effect of education and training on RRP, (5) checking for reproducibility, and (6) responsible and fair peer review. These themes have in common that they address aspects of research that are mostly on the level of the scientific system, more than on the level of the individual researcher. Some current initiatives are already gathering substantial empirical evidence to start filling these gaps. We believe that with sufficient support from all relevant stakeholders, more progress can be made.

## Introduction

While academic research is generally recognized as an institute of crucial importance to the well-being of our contemporary society, concerns over its functioning are growing. The current coronavirus disease 2019 (COVID-19) pandemic especially highlights the need for more open and collaborative research practices. Stakeholders from within and outside the academic community are increasingly worried about issues regarding research funding, the scientific publication system, research evaluation practices, and academic career trajectories. Fueled by several highly visible cases of scientific misconduct—both before the COVID-19 outbreak and during the current pandemic (Callaway, 2011; Mehra et al., 2020)—there is a growing awareness that the results of scientific research should be reliable, that research practices should be responsible, and that the workflows and results of research need to be transparent. While media coverage has mainly centered on large fraud cases, the research community has acknowledged that fundamental causes of current issues are embedded in more systemic aspects of evaluating, rewarding, and disseminating research (Biagioli et al., 2019; Bramstedt, 2020; Fleming et al., 2020). Experts of research integrity are convinced that, on an aggregate level, factors such as detrimental research practices, lack of supervision and mentoring, the system of research with hypercompetition, unidimensional assessment criteria, an individualistic research culture, and publication pressure are more threatening to the reliability and validity of research than (arguably rare) cases of misconduct (Begley & Ioannidis, 2015; Fanelli et al., 2015; Martinson et al., 2010). This has led to a shift in focus towards research on responsible research practices (RRP). The increased attention for this subject is reflected by the large number of international initiatives from various stakeholders to foster RRP (e.g., The Research on Research Institute, the Meta-Research Innovation Center at Stanford [METRICS]). These initiatives have mainly emerged from the biomedical sciences and psychology, and there is now an increasing need for initiatives tailored to other research disciplines and cultures (Haven et al., 2019b; Horbach & Halffman, 2019).

Research on RRP has shown several trends. Initial interest in this field was mainly in defining research integrity and gaining more insight in the different forms of research misbehaviors, leading to classifications of major and minor forms of misbehaviors (falsification, fabrication, and plagiarism and questionable research practices [QRPs], respectively; [Bouter et al., 2016; Steneck, 2006]). In addition, ample attention has been paid to estimating the frequency of transgressions and analyzing individual cases (Fanelli, 2009; John et al., 2012; van der Heyden et al., 2009). Repeatedly, boundaries between research integrity and research ethics have been questioned in this debate with other concepts such as responsible research and innovation prominently coshaping discussion on research integrity. This has led us to adhere to a broad interpretation of “research integrity” including aspects that some may classify as research ethics. The scope of our concepts and paper will be further clarified in the methods section, which is added as Supplemental file Appendix I.

Over the past years, research on RRPs and research integrity in all disciplinary domains is increasingly redirecting its focus towards root causes and possible solutions. This has resulted in a shift from microlevel analyses, mainly concerned with the individual and his/her characteristics, towards current discussions acknowledging the important role of broader cultural, organizational, and systemic factors, including the research climate, organizational settings, and incentive structures (Haven et al., 2019b; Martinson et al., 2010). As an example, this shift is represented in the new Dutch Code of Conduct for Research Integrity, dedicating a full section to institutional responsibilities (KNAW, 2018).

In addition, openness and transparency have been increasingly identified as drivers of RRP. A growing number of initiatives are calling for and facilitating increased transparency in research, voiced in terms like “FAIR Data,” “Open Access,” and “Open Science.” Initiatives include proposals as diverse as calls for openly sharing data, facilitating reproducibility and replication studies (including the humanities), publishing in Open Access journal articles, and using Open Peer Review formats (Nosek et al., 2015). Lastly, suspicion about published findings that are not replicable, mainly within psychology and the biomedical sciences, has directed research focus of RRP researchers towards ways of enhancing replicability. This includes studying publication bias, methodological flexibility, and transparency (Munafò et al., 2017; Wicherts, 2017).

While the number of initiatives aiming to foster RRP is growing and diversifying, the evidence base for which initiatives are actually successful is often lacking. In addition, initiatives are commonly restricted to specific niches or academic disciplines. In particular, most initiatives and studies originate from the biomedical and social sciences, leaving blind spots in other research disciplines, cultures and methods, and risking the tendency to overgeneralize both challenges of research integrity as well as their potential solutions. Therefore, more research is still needed to guide the implementation of RRP across the full range of academic disciplines, cultures, and settings.

This paper aims to shed light on the international debate on RRP addressing three key issues:

1. A sketch of the current international discussion on RRP.
2. An overview of current initiatives and already obtained results regarding RRP.
3. An overview of potential future needs for research on RRP.

Our opinion piece will hence address these three points. Its ultimate aim is to serve as a starting point for future research agendas and more broadly for the international research field on research integrity and RRP. However, the current document is slightly focused on the Dutch context. This is partly due to the fact that the current debate on responsible research in the Netherlands was sparked by the high-profile fraud case of Diederik Stapel. Stapel was a professor of social psychology who fabricated data in over 50 published articles. The case itself triggered a plethora of Dutch initiatives to foster RRP (Abma, 2013). This also included initiatives from funding agencies such as the European Union in their H2020 program, the Office of Research Integrity in the United States, the funder ZonMw in the Netherlands, and the Welcome Trust in the United Kingdom, establishing multiple funding programs (e.g., [Replication Studies, 2019; Research on Research, 2020]). In these programs, several researchers are currently working on and assessing initiatives that foster RRP. Although we have tried to incorporate an international perspective, we found several initiatives from the Netherlands. Since the Dutch health funding organization was one of the first funding organizations that started funding on research integrity and numerous initiatives have been initiated in the Netherlands, the Netherlands can be considered one of the frontrunners in the area of initiatives and research fostering RRP, we believe this focus constitutes an appropriate lens to inventories state-of-the-art developments in the field. The progressive atmosphere in the Netherlands regarding research and initiatives on RRP is among others exemplified in several grant schemes by national funders related to this topic, as well as the early establishment of a national committee on research integrity. In our endeavors to establish a research agenda, we first summarize the current state of research on RRP and provide a conceptual overview of initiatives to foster RRP. Second, we identify major gaps of knowledge in the field of research on RRP that need to be addressed. This builds to a research agenda in which we classify the most pressing themes that need further study. With this knowledge, we aim to inform policymakers and funders on current issues for RRP. The paper thereby complements recent initiatives that have inventarised research on specific aspects related to RRP, such as peer review and research integrity (Aubert Bonn & Pinxten, 2019; Tennant & Ross-Hellauer, 2020).

## Current Initiatives Promoting RRP

The scientific community has already started many initiatives to promote and address RRP. These initiatives cover all aspects of the scientific “empirical process” and are in differing stages of development and acceptance by researchers. In this section, we categorize the main types of initiatives and sketch the current state-of-the-art of RRP. A list of concrete examples of initiatives can be found in Supplemental file Appendix II, whereby we acknowledge that the list is not exhaustive and may overlook other initiatives.

**Figure 1.**
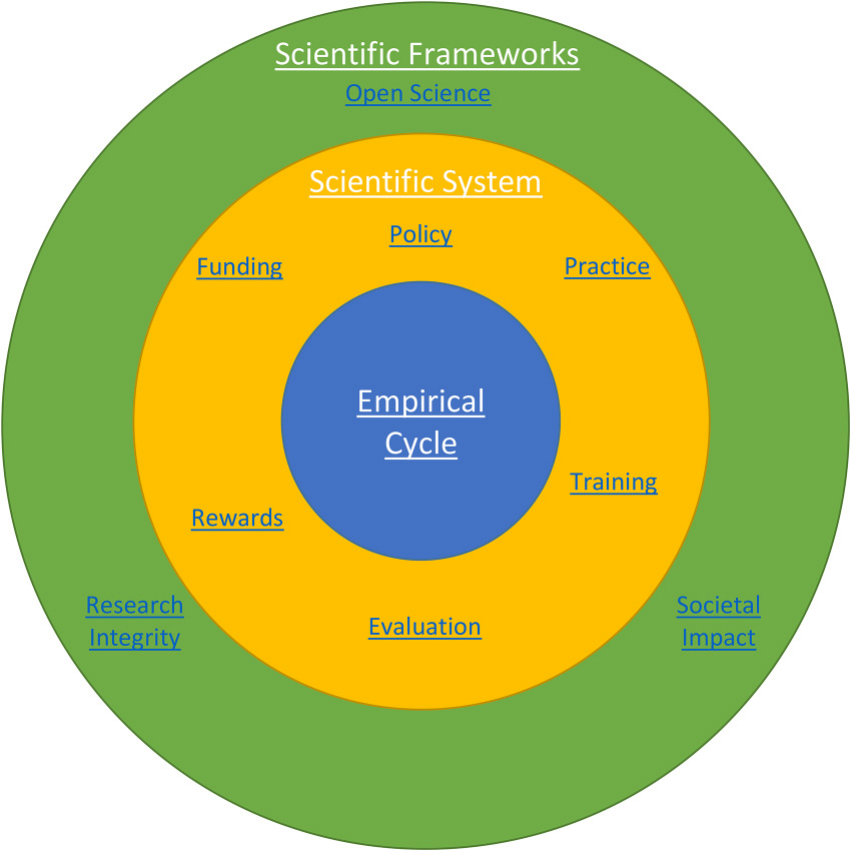
A schematic representation of the elements that interact in responsible research practices (RRP). The three elements all consist of initiatives that we have collected in Supplemental file Appendix I. The three rings in this figure represent the different levels at which we can look at RRP: scientific frameworks, the scientific system, and the empirical cycle. Figure 2 elaborates on the initiatives to promote RRP in the empirical cycle.

RRP effectively encompasses the entire scientific ecosystem from overarching frameworks to the implementation system to the research process (Mejlgaard et al., 2020). In Figure 1, we have framed RRP as an overarching theme consisting of these three different levels: scientific frameworks; the scientific system; the empirical cycle. We are aware that we focus on research adhering to a research paradigm following the empirical cycle and thus put less emphasis on other methodologies. Other ways to structure our data could have been chosen, for instance a framework based on Latour and Woolgar's credibility cycle. Our choice for structuring the data along the empirical cycle is not so much because we believe it is the best way to describe research in general, but because we believe it is most appropriate for our current task as most initiatives are tailored to a research paradigm fitting this cycle (Latour & Woolgar, 1979). We acknowledge that some disciplines may not be fully covered by this paradigm. In fact, we will address this as one of the major shortcomings of previous research on RRP. Nevertheless, we believe that the empirical cycle is a useful framework to create and present an inventory of current initiatives that aim to foster RRP.

### Scientific Frameworks

The overarching first level of scientific frameworks refers to three broad value frameworks under which all RRP are expected to be carried out and judged in the current socio-political climate: open science; research integrity; societal impact. These three frameworks are not to be seen as distinct but rather overlap and feed into each other. In fact, by making research more open and transparent, open science is expected to be an enabler for research integrity and societal impact (Moher et al., 2020).

The practice of an open science has existed for centuries in Europe in the form of open letters and select publications in scholarly outlets. A modern interpretation of *open science* has been proposed by the European Commission (2016) as the opening up of research workflows and outcomes via digital technology. This approach applies to the entire research process: designs and methodologies (open methodologies); data that is findable, accessible, interoperable, and reusable (www.go-fair.org) and open (open data); software code (open source); initial versions of publications and peer review (open peer review); and access to final versions of publications (open access). This approach also includes opening up study materials (open education) and involving citizens in research (citizen science). The European Union has taken a leading role in open science with *Plan S* (www.coalition-s.org).

The concept of *research integrity* is crucial to the scientific endeavor and refers to research practices that follow five key principles of honesty, scrupulousness, transparency, independence, and responsibility (see the European ALLEA code and Netherlands Code of Conduct for Research Integrity [European Code of Conduct for Research Integrity. Revised edition, 2017; KNAW, 2018]). Research misconduct arises from researchers intentionally or unintentionally not following these principles. High-profile cases of intentional misconduct often lead to public outcry and calls for changes in the way we do science. Examples of research integrity initiatives in the Netherlands are the Netherlands Research Integrity Network (www.nrin.nl) and the EU-funded Embassy of Good Science (www.embassy.science).

Similarly, there has been considerable public indignation about the (lack of) *societal impact* of publicly funded research. There is a growing call for research activities and outcomes to not only involve citizens but also be relevant for and benefit society. This can take many forms: opening up and communicating about the outcomes of research; direct application and commercialization of research technologies; directly involving the public in the design, collection, and analysis of research; or directly letting the public decide future research funding topics. The Netherlands aims to facilitate this by the participatory National Research Agenda (www.wetenschapsagenda.nl).

### The Scientific System

The secondary level of the scientific system focuses on the steering and facilitation of RRP by governments, funding agencies, and academic institutions under six key areas: research policies; research practices; training researchers; evaluating research(ers); rewarding researchers; funding research(ers). Each of these areas may focus on specific aspects of the scientific framework as well as individual steps in the empirical cycle. We are convinced that one of the goals of the scientific system for RRP is to ensure that researchers learn about RRP and conduct their research in a “responsible” manner.

The *policies* for RRP relate to the legislation affecting research, funding programs, guidelines and codes of conduct, and general principles for how researchers are to be trained, evaluated, and rewarded. These policies crucially determine the initiatives that are intended to foster RRP. Any identified gaps in initiatives should lead to new policies for stimulating initiatives to fill such gaps. This position paper aims to identify any gaps for developing new policies. Research performing organizations (incl. universities) strongly determine which policies and governance structures are shaping the research environment individual researchers work in. This responsibility is oftentimes overlooked. This research environment is influencing the behavior of researchers (Haven et al., 2021) and is even perceived as an important issue that may trigger research misconduct (Davies, 2019). We would like to emphasize the crucial role and responsibility research performing organizations have to foster research integrity. The European SOPs4RI project has already started collecting tools to help institutions foster RRP (Mejlgaard et al., 2020). Aside from formal policies, actual research *practices* form the most effective initiatives to foster responsible research. While stakeholders agree that mere changes in policies, guidelines, and codes of conduct will not suffice to establish more responsible research, much attention has been given to these formal academic structures. Nonetheless, actual research practices, as well as informal rules, academic cultures, and interpersonal relations have been the target of recent initiatives and studies on RRP.

The raising of awareness and *training* of researchers in RRP is crucial for RRP to be carried out successfully and be widely adopted by the scientific community. A recent survey by the European Commission (O'Carroll et al., 2017, See Appendix 3 for the survey results which show that researchers have little knowledge of Open Science), however, has shown that the majority of researchers in Europe do not fully understand and are not being supported to implement the principles of open science. Initiatives for supporting and training researches in RRP are currently grassroots and locally organized, such as the Superb Supervision (https://amsterdamresearchclimate.nl/superb-supervision) course or the ReproducibiliTea journal clubs (www.reproducibilitea.org).

The *evaluation and rewarding* of research and researchers are perhaps the most important factors in promoting RRP. The current evaluation system is focused on judging research, and by extension researchers, according to the number of publications that appear in high impact factor and branded journals, instead of judging the quality of the research itself. Furthermore, researchers are to a much lesser extent (if at all) evaluated on the basis of other research-related activities such as teaching, supervision, unsuccessful grant applications, research designs, peer reviews, the publishing of datasets, and public engagement. These “perverse incentives” (Bouter, 2015) are exacerbated by the high publication pressure and strong competition over research funding and job opportunities that researchers experience (Martinson et al., 2009; Tijdink et al., 2014; van Dalen & Henkens, 2012). Examples of initiatives that aim at changing this incentive structure are the Declaration on Research Assessment (www.sfdora.org) and the Hong Kong Manifesto (Moher et al., 2020).

Finally, the *funding* system drives academic research and is also closely related to the careers of researchers. The funding system has the potential to directly influence the behavior of researchers via funding mandates and funding award criteria. One example is the mandating by funders of researchers to publish in Open Access journals and openly license their publications. Another example is the refusal by funders to use journal impact factors to evaluate researchers: this reduces the pressure on researchers to publish only in high-impact and branded journals. Funding therefore directly determines the research agenda of researchers and can be more aware of their crucial role in fostering RRP.

### The Empirical Cycle

**Figure 2.**
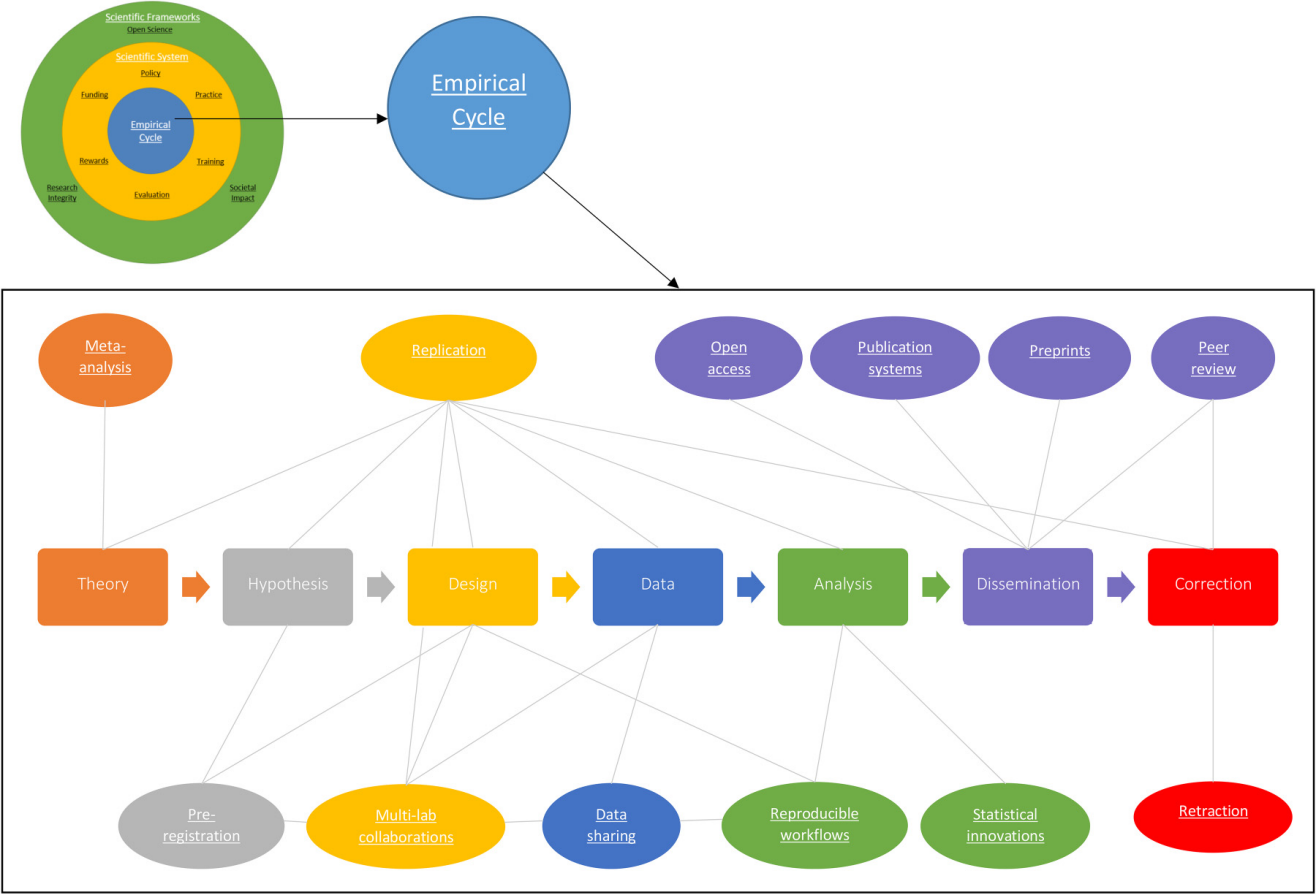
Schematic overview of the current initiatives to promote responsible research practices at the level of the empirical cycle. The initiatives (in the ellipses) are linked to different elements of the empirical cycle (rounded squares) and to each other with the grey connector lines. Each underlined term links to a list of concrete initiatives in Supplemental file Appendix I.

Most of the initiatives to promote RRP are aimed at the empirical cycle itself. Figure 2 shows the many types of initiatives at this level, including how they link to the different steps in the empirical cycle and how they relate to each other. In many fields and types of research, the empirical cycle often roughly follows the same steps. From a theory (step 1), a hypothesis is formulated (step 2). To test this hypothesis, a study is designed (step 3) and data are collected (step 4). Based on the analysis of the data, a conclusion is drawn (step 5) and the research is disseminated (step 6). Finally, during and after dissemination, the published literature can be corrected (step 7). These steps are loosely based on the empirical cycle as discussed by De Groot (1961). We realize that not every single scientific study will fit this representation (e.g., exploratory research where the data generates the hypothesis or conceptual research fields that do not employ data). However, most of the initiatives to promote RRP at the empirical level seem to fit within this framework. The fact that most initiatives fit this framework is also indicative of a potential lack of diversity in those initiatives, arguably not covering research methods and traditions working from different frameworks. In the paragraphs below, we briefly outline the initiatives to promote RRP at each of these steps in the empirical cycle. Furthermore, for each step and each practice we have collected a list of initiatives that fall under these practices. The full list is available in Supplemental file Appendix II.

In the first step of the empirical cycle, we emphasize the need for theory. To build theory or to assess which findings are sufficiently robust to build upon, researchers usually aim to compile results from existing studies. One method to synthesize results is via *meta-analysis*: a statistical summary of different studies with (more or less) the same research question.^1^ A problem with meta-analyses is that they are not immune to problems such as publication bias, which means that they can lead to biased results.

In the next step of the empirical cycle, a hypothesis is formulated. At this step, one of the most notable types of initiatives to increase RRP is preregistration (Kupferschmidt, 2018). There are different ways to preregister a study, but generally, researchers publish their hypotheses and research plan online, before conducting the study. Preregistration can have several goals. First, it allows for a clear distinction between confirmatory and exploratory analyses (Wagenmakers et al., 2012). Second, it could prevent exploiting flexibility in methods and data analyses to obtain the desired result, because the plans were registered beforehand (initiatives such as Compare [www.comparetrials.org] and EU Trials Tracker [eu.trialstracker.net] compare registrations with the accompanying published papers). Third, preregistration could decrease the effects of publication bias. The practice of preregistration has been standard in biomedicine for years, but has only recently gained popularity in the social sciences. One relatively new form of preregistration is a “Registered Report” (Chambers et al., 2015). Here, the preregistration is submitted to a journal and peer reviewed. Once the preregistration is approved, the authors can get an “in principle acceptance,” meaning that if they follow their preregistration, their paper will be published regardless of the outcome. Over 260 journals now accept this format.

The next phase in the empirical cycle is the study design phase. Two notable types of initiatives that promote RRP at this step are *multilab collaborations* and *replication studies*. Multilab collaborations are coordinated efforts to run the same study in different labs, sometimes across the world (see the Psychological Science Accelerator [psysciacc.org] and the Many Babies project [https://psyarxiv.com/27b43/]). This strategy increases statistical power and allows investigating the generalizability of the findings. It also requires detailed study protocols, which increase reliability. A second initiative to improve RRP at the design step is a stronger focus on replication studies. Replications affect the design phase, but they also link to many other steps in the empirical cycle; from theory to analysis. An example of a Dutch initiative to promote replication studies is the replication grant of NWO (Replication Studies, 2019). A special case of a replication study is a “registered replication report”: a multilab collaboration that performs a preregistered replication study. Another example of fostering responsible collaboration is the Montreal statement on research integrity in cross-boundary research collaborations (Lancet, 2013). This statement highlights the importance of equitable institutional cross-boundary collaborations.

Another clear point in the empirical cycle where RRP can be stimulated is when data has been collected. One of the most advocated strategies is to *share data*. Openly sharing data allows for reanalysis to detect and correct mistakes, to check robustness, and to answer new research questions. Several stakeholders (journals, funders, institutions) now require Open Data, and there is an increasing number of infrastructural solutions to facilitate data sharing. One crucial prerequisite for opening data, as noted earlier, is that the data is made FAIR (www.go-fair.org). Data that has been made FAIR can, but may not necessarily, be opened afterwards.

In the analysis step of the empirical cycle, RRP are promoted via initiatives related to *statistical innovations* and *reproducible workflows*. Statistical innovations include rethinking thresholds for statistical significance, advanced analyses, and promoting statistical frameworks (e.g., frequentist vs. Bayesian statistics and effect size estimation). An example is the development of the free statistical software such as JASP (jasp.stats.org) and R (www.r-project.org). Most of these innovations focus on increasing the reliability and robustness of statistical conclusions. Other initiatives focus on increasing the reproducibility of workflows. A study is reproducible^2^ if a reanalysis of the data, following the reported procedures, leads to the same results. Reproducibility requires that data should be available and that procedures should be clear. Reproducibility can be greatly improved if researchers manage their data according to the FAIR data principles. Researchers can share their data via online data repositories, such as the Dataverse Project, Figshare, or Dryad. Furthermore, platforms such as the Open Science Framework (www.osf.io) can serve as a data repository. Another initiative that highlights the importance of reproducibility is the introduction of “Verification Reports” (Chambers, 2020) in the journal Cortex. The sole purpose of this new type of publication is to assess the reproducibility of previously published papers.

To guarantee reproducibility of systematic reviews and meta-analyses, more and more journals require authors to follow specific reporting guidelines (see also *policy*), such as the CONSORT statement for randomized controlled trials, and PRISMA or MARS for meta-analyses and/or systematic reviews. These guidelines serve as checklists for authors to make sure that all important information is included in their article. They result in more standardized reporting, which makes it easier for researchers to find the information they need, and to verify results and conclusions.

The next step of the empirical cycle is dissemination. In most cases, this entails publishing a study in a peer-reviewed journal. Many initiatives promoting RRP appear to focus on this step. Consider, for instance, initiatives that shift the focus to prepublication peer reviews, by promoting *preprints*, published online in preprint archives (e.g., Biorxiv.org). Yet other initiatives aim to revise the publication system completely via innovative online platforms (e.g., the megajournal F1000; www.F1000research.com).

The final step in the empirical cycle is the correction of research reporting. Correction can take place before/during dissemination through peer review. Correction can also take place after dissemination through replication (for initiatives linked to replication, see above), and errata or retractions (see, e.g., the popular blog Retraction Watch [www.retractionwatch.com] that tracks scientific retractions and covers cases of misconduct).

## Themes That Warrant Future Research

Before we start our discussion on themes that warrant future research, we would like to stress that we consider responsible research as the product of applying the overarching scientific frameworks of open science, research integrity, and societal impact across the scientific system and in the empirical cycle. This is a concern for the entire research enterprise, and for all scientific domains, even though it may manifest itself differently in diverse research disciplines. Currently, these differences are poorly understood. The traditional focus on several disciplines is reflected in the list of abovementioned initiatives, stemming mainly from the biomedical and some of the social sciences, leaving the natural sciences, engineering, humanities, and the other social sciences understudied. Some studies have already indicated how integrity challenges emerge differently across research disciplines (Haven et al., 2019a, 2019b; Horbach & Halffman, 2019), but future studies should amplify the current initiatives towards other disciplinary domains and put more efforts into studying and resolving these differences.

While the above list of current initiatives regarding RRP consists of laudable efforts, and sketches a hopeful picture, much remains to be elucidated. Many of the initiatives described are relatively understudied and there remains a paucity of evidence on their effectiveness and potential impact. Few controlled studies have compared the differences between new initiatives with existing frameworks, as well as the potential hurdles and consequences of implementing the initiatives in different research settings.^3^ This could be a theme that warrants future research in itself.

In this section, we will outline some of the most prominent knowledge gaps that might be effectively addressed in future funding programs addressing RRP.

Our themes emerged from a systematic approach. First, we created an overview of current initiatives concerning RRP across different scientific disciplines. We clustered these initiatives in a thematic map to identify any gaps. Based on this overview, we created a set of underrepresented themes in the current research on RRP and condensed this set into a list of six major themes that we believe lack a robust evidence base and that can be effectively addressed in future funding programs. We asked 16 experts in the field of meta-research and/or RRP for feedback on the identified themes and their rationale. Below, you will find a summary of the six most important themes. The full list of themes can be found in Supplemental file Appendix III. More information on the methodology section can be found in the Supplemental file Appendix I.

First, we believe the *responsible evaluation of research and researchers* is a crucially understudied issue. The current evaluation criteria are thought to create a perverse incentive structure, are unidimensionally focused on the “bean counting” of publications in high-impact and branded journals, and may nudge researchers unconsciously into QRP with a focus on publishing as many articles as possible, instead of getting it right. How these incentive structures actually influence research practices is still unknown and how the research evaluation criteria should be reformed needs further research. The urgency for this research was also voiced during the 6th World Conference on Research Integrity (WCRI2019) in Hong Kong in June 2019. Research on this theme may also shed light on the obstacles to address. Finding out the reason for the lack of uptake could help form better policies and incentives. Furthermore, we recommend formal consideration by funders of ways to offset risks to early-career researchers in engaging in open research practices and ensuring proper reward and incentive structures are in place (e.g., grants and fellowship schemes that take into account commitment to open practices).

Second, we believe that the *influence of open science and transparency on RRP* requires further study. The open science movement is quickly adopting various initiatives that help to create a more open and transparent science (sharing data, preregistering studies, and openness in peer review and in publishing). While researchers may agree that open science can benefit RRP, there is much misunderstanding on what “open science” actually means. More research is thus needed to address the possible benefits and disadvantages of this trend in the research community. We are also still unable to determine if preregistration and the sharing of data are reliable determinants that reduce publication and outcome reporting bias. A shift to open science will furthermore require a coherent operationalization of the many open practices across the empirical cycle so that researchers can work effectively and efficiently in an open manner. The relationship between open science and innovation, especially in collaborations between academic institutions and industry where openness can hamper innovation, is lastly understudied and needs further scrutiny. A shift to open science and increased transparency should also involve reducing the bias of solely publishing positive research: negative and null results are just as important for advancing science.

Third, we identified a lack of knowledge on *research on responsible mentoring, supervision, and role modeling.* Mentors and supervisors play a key role in establishing a responsible research climate for early-career researchers. As such, they have an enormous influence on the next generation of researchers and thus on the (future) practice of responsible research. Furthermore, most of the education in crucial phases of an academic career happens through socialization processes, largely influenced by mentors, supervisors, and role models. However, this pivotal role is not always fully acknowledged and it is seldom thoroughly reviewed in academic research settings. The role of bad supervision or even harassment can furthermore have detrimental effects on research and researchers. We believe that additional research may shed light on this, which will eventually assist in mentors and role models establishing a research culture that fosters RRP.

Fourth, the *effect of education and training on RRP* needs further study*.* The role of education and training is commonly proposed as one of the main interventions to foster RRP. However, the research on its effectiveness is inconclusive and restricted (Marusic et al., 2016). Besides, hardly any educational programs are organized with a focus on responsible research in senior researchers. Empirical research should assess what type of education and training is successful and describe what potentially or definitively can make a difference.

Fifth, future research programs should focus on increasing the *reproducibility of their workflow and analyses*. We need to investigate how we can facilitate and incentivize sharing data, analysis scripts, protocols, and other relevant materials that are necessary to retrace the steps that the original researchers took to reach their conclusion. More research could expose possible factors that limit researchers to reflect on the reproducibility of their results and make people aware that reproducibility, at least in many research disciplines and cultures, is one of the pillars of RRP.

Finally, *responsible and fair peer review* is currently understudied and does not receive sufficient attention in research funding programs. Peer review is often considered to be one of the cornerstones of academic research. Currently, it is still largely a black box at the risk of conflicts of interest, unfair procedures, serendipity, and inconsistency. This leaves questions such as: How can we improve peer review? What role can transparency play? Do we know enough about peer review to propose novel strategies and interventions that can make peer review more reliable? Can responsible peer review reduce publication bias? What can automated software do to alleviate the enormous pressure on the peer review system? More research can detect potential flaws and can search for novel techniques that help us to improve the peer review process, ultimately making it more efficient and trustworthy.

For all these themes we believe that it is crucial to acknowledge the epistemic, methodological, gender, and cultural *diversity* in research in order to comprehensively tackle issues of QRP. In this light, we think that different paradigms should be open and transparent and include alternative perspectives to look further, to expand thinking and to initiate collaborations with other paradigms. Relating to researchers with diverse foci in their solution approach to breaches of research integrity, for example, those focusing on methodological fixes to enhance reproducibility and replicability, and others focusing on open science initiatives (Murphy et al., 2020), our research agenda emphasizes that those approaches should be harmonized and should move away from the one-size-fits-all solutions. To move away from one-size-fits all approaches to solving these issues, we need to better understand which QRP are prevalent in the different academic disciplines and which RRP are most effective in these disciplines. For the themes mentioned above, this means for example that training, mentoring, and supervision should be tailored to the specific needs of a community, allowing different approaches across different disciplines and academic ranks.

## Conclusion

In conclusion, we aimed to systematize and categorize the initiatives that foster and study RRP. We have mapped these initiatives and subsequently identified gaps of knowledge and underrepresented themes in RRP that we believe require further exploration. This can serve as a starting point for research agendas on RRP.

We have identified six main themes for further attention: responsible evaluation of research and researchers; the influence of open science and transparency on RRP; research on responsible mentoring, supervision, and role modeling; the effect of education and training on RRP; checking for reproducibility; and finally, responsible and fair peer review.

The themes that we find underrepresented are broad areas of research that focus on the level of the scientific system, rather than the microlevel of individual researchers’ research practices and the associated QRPs. Some current initiatives are already gathering empirical evidence to start filling these gaps. However, we do feel that future funding programs should take these gaps into account in order to bring the field of research on research integrity a step further.

We lastly recognize the complexity of the scientific ecosystem as well as diverging interests of stakeholders within that ecosystem. Some stakeholders have nonscientific, for example, professional, financial, or commercial incentives for maintaining the status quo and will not necessarily be open to change. This includes academic publishers that generate their revenue from subscription and paid open access models, university libraries that aim to secure the best access and publishing deals, and senior academics who owe their positions and status to the current recognition and rewards structure. The road to improving RRP should ultimately involve alignment across all key RRP stakeholders, which requires a sensitivity to stakeholders’ background and interests and requires a unique and theme-specific approach across our emerging themes that warrant further study.

In conclusion, we feel that there is an urgent need for more research on research and research practices. While the focus in our empirical material has been slightly biased towards the Dutch context, we feel it sufficiently represents the global state of affairs. Specifically, studies of RRP and how to foster them require additional support in a global context. Both the current academic debate on these issues, as well as wider societal concerns, are indicative of this urgency. We moreover believe that current initiatives have made important steps in the right direction already, and with sufficient support from various stakeholders, more progress can be made. We hence call upon all relevant stakeholders to actively engage in efforts to further support studies on RRP and initiatives to foster them.

## Supplemental Material

sj-docx-1-jre-10.1177_15562646211018916 - Supplemental material for Towards a Research Agenda for Promoting Responsible Research Practices Click here for additional data file. Supplemental material, sj-docx-1-jre-10.1177_15562646211018916 for Towards a Research Agenda for Promoting Responsible Research Practices by Joeri K. Tijdink, Serge P.J.M. Horbach, Michèle B. Nuijten and Gareth O’Neill in Journal of Empirical Research on Human Research Ethics

## Notes

1. Researchers with a qualitative focus typically produce a systematic review involving the collection, critical appraisal, and synthesis of all relevant studies.

2. Please note that we use the term *reproducibility* to refer to analytical reproducibility, which is not the same as replicability. Replication and replicability refer to a situation in which new data is collected to assess whether the original results can be *replicated* in a new sample.

3. However, we do note the rise of “meta-scientific” studies and even entire meta-research groups (e.g., the Meta-Research Center (www.metaresearch.nl) at Tilburg University and METRICS at Stanford University; metrics.stanford.edu) that study exactly these questions.
