# Supplementary material for: Towards a Research Agenda for Promoting Responsible Research Practices
Source: J Empir Res Hum Res Ethics. 2021 May 26;16(4):450–60. doi: 10.1177/15562646211018916 (PMC8458678; doi:10.1177/15562646211018916)
Supplement: sj-docx-1-jre-10.1177_15562646211018916 - Supplemental material for Towards a Research Agenda for Promoting Responsible Research Practices [file sj-docx-1-jre-10.1177_15562646211018916.docx]

# Appendix I

# Methodology

This position paper emerged from the close collaboration of its authors in several steps. The authors were chosen for their differing expertise in the field of RRP and by the fact that they are early-career researchers, an important group which will not only have to adjust to future RRP but form the future generation of senior researchers. For more information on the authors, see the biographies at the end of the paper. The authors drew initially on their own academic background, expertise, academic networks, and literature reviews to establish an informed advice. Through various forms of wider consultation, we then aimed to align our personal perspectives with those in the field of research on RRP and to solicit input from multiple stakeholders with their varying stances to gain a broader consensus.

*Step 1: Exploratory phase*
After an initial round of discussion among the authors, each author individually provided an overview of current initiatives and underrepresented topics in the field of RRP. These overviews were inspired, but not restricted to, the personal background and knowledge of the authors, supplemented by discussion within their peer networks. While this paper does not provide a systematic review of the academic literature on RRP, it is inspired by literature reviews by the individual authors.

*Step 2: Creating an overview of current initiatives across different academic disciplines*
In a second stage, the independent overviews were combined and supplemented to form one set of current initiatives and studies focusing on RRP. The initiatives were then collaboratively clustered into a thematic map of current initiatives, showing networks, connections, and dependencies between the various themes. The results of this endeavour are presented in section 3 of this paper.

*Step 3: Identifying underrepresented themes*
In a third phase, the underrepresented topics identified by the individual authors in step 1 were combined to form a list of themes that are in need of further research. This list encompasses important gaps in knowledge on RRP that need more attention in future funding programmes. This list was established through discussions on the scope of our project and the characteristics of the identified gaps, combining similar themes and deleting themes out of scope.

*Step 4: Creating a ranking of underrepresented topics*
We subsequently created a ranked list of the underrepresented topics from step 3. This was done in a two-tier manner. Each author independently ranked the complete set of underrepresented themes through a short ranking exercise that made it possible to collect the results of the ranking exercise from each author. We discussed the main underdeveloped topics in light of the ranking exercise and reached consensus by discussion among the authors on which topics should be emphasized with recommendations for future funding programmes. Each author was then asked to provide a brief justification for their prioritisation of the top five themes in need of further research. The authors discussed which of the themes currently lack a robust evidence base and could be effectively addressed in future funding programmes. The resulting rankings were subsequently merged into a single ranking by calculating the overall ranking scores of the authors. The resulting ranked list of underrepresented research topics is presented in section 4 of this position paper.

*Step 5: Drafting the paper, based on the results of steps 1-4*

*Step 6: Consultation of first draft by 16 experts in the field of RRP.*A list of experts in RRP was assembled and all experts were then asked to provide feedback on the draft position paper. All expert suggestions were taken into account and, as far as possible, incorporated into the position paper. The contributing experts are listed in the [Acknowledgements](#_7._Acknowledgements).

*Step 7: Recommendations for future funding programmes on RRP*

# Appendix II: Examples of Current Initiatives to Promote Responsible Research Conduct

This list contains examples of current initiatives to promote responsible research practices. Please note that this list is not exhaustive, and mainly serves as an illustration. For a schematic overview how all these initiatives relate to each other, see Figures 1 and 2 in the main text. Based on the framework we introduced in Figure 1, we discussed the examples of initiatives at each of the different levels that we were aware of. Furthermore, we have used the results from the INSPIRE project (see [here](https://embassy.science/wiki/Theme:3c6a13ad-6861-4a5f-bf5b-491693ee6b6d)). The INSPIRE project, which stands for 'Inventory in the Netherlands of Stakeholders’ Practices and Initiatives on Research integrity to set an Example', aims to collect, classify and share international initiatives to foster research integrity. The project's ultimate goal is to inspire and enable others to implement similar initiatives. Such initiatives include training courses, policies, campaigns, consultations etc. From their inventory, we have picked the examples that we found most exemplary for our manuscript.

## Scientific Frameworks

### Open Science

- Open Science [Amsterdam Call for Action on Open Science](https://www.government.nl/documents/reports/2016/04/04/amsterdam-call-for-action-on-open-science)
  *Conference in Amsterdam in 2016 that set goals for an Open Science agenda in Europe*
- [National Plan Open Science](https://www.openscience.nl/en/national-platform-open-science/national-plan-open-science)
  *National strategy proposing concrete steps to achieve Open Science in The Netherlands*

### Research Integrity

- [Embassy of Good Science](https://www.embassy.science/)
  *A place where the community can share experiences and insights, deepening understanding and continuously contribute to the development of good science.*
- [Netherlands Research Integrity Network](https://www.nrin.nl/)
  *A Dutch network that aims to facilitate collaboration, exchange and mutual learning between the actors in the field of research integrity*
- [Retraction Watch](http://retractionwatch.com/)
  *Popular blog tracking scientific retractions and covering cases of misconduct.*

### Societal Impact

- [Knowledge Transfer Offices (KTOs) and Technology Transfer Offices (TTOs)](https://www.universityinnovation.nl)
  *Offices at universities that contact research and innovation stakeholders to valorise research*
- [National Science Agenda](https://wetenschapsagenda.nl/)
  *Dutch Initiative aiming, among others, to enhance societal relevance of research by inviting the wider public to contribute to research at various stages.*
- [Standard Evaluation Protocol (SEP)](https://www.knaw.nl/nl/actueel/publicaties/standard-evaluation-protocol-2015-2021)
  *Dutch protocol for assessing research and education that includes societal impact criteria*

## The Scientific System

### Policy

- [Fostering Responsible Research Practices (BVO)](https://www.zonmw.nl/en/research-and-results/fundamental-research/programmas/programme-detail/fostering-responsible-research-practices)
  *Research funding programme in The Netherlands that aims to foster responsible research*
- [Science in Transition](https://scienceintransition.nl/)
  *Movement to improve the rewarding and societal relevance of research in The Netherlands*
- Journal reporting guidelines
  - [CONSORT](https://apastyle.apa.org/manual/related/moher-2001.pdf)
    *Reporting guidelines for randomized controlled trials*
  - [PRISMA](http://www.prisma-statement.org/), [MARS](https://wmich.edu/sites/default/files/attachments/u58/2015/MARS.pdf)
    *Reporting guidelines for meta-analyses and systematic reviews*

### Practice

- [Society for the Improvement of Psychological Science (SIPS)](http://improvingpsych.org/)
  *SIPS is a service organization aimed at bringing together scholars working to improve methods and practices in psychological science.*
- [BITSS](https://www.bitss.org)
  *The Berkeley Initiative for Transparency in the Social Sciences aims to enhance the practices of economists, psychologists, political scientists, and other social scientists in ways that promote research transparency, reproducibility, and openness.*
- [Implementation ZonMW’s BVO](https://www.zonmw.nl/en/research-and-results/fundamental-research/programmas/programme-detail/fostering-responsible-research-practices/)
  *ZonMw is currently funding several implementation projects that aim to translate findings from the BVO programme into actual interventions that foster responsible research practices.*

### Training

- [Dilemma Game](https://www.eur.nl/over-de-eur/strategie-en-beleid/integriteit/wetenschappelijke-integriteit/dilemmaspel) *Card game on research integrity developed by Erasmus University, inviting players to deliberate on dilemmas and potential ways of dealing with them.*
- [FOSTER Open Science Portal](https://www.fosteropenscience.eu) *Online portal for training researchers in Open Science practices via courses and webinars*
- [On Being a Scientist](https://www.youtube.com/watch?v=tCgZSjoxF7c&feature=youtu.be) *Short, professional movie describing several research integrity challenges in context*
- [Open Science Communities](https://openscience-utrecht.com) *Grassroots network of researchers at Dutch universities to teach and support Open Science*

- [PRINTEGER](http://printeger.eu/) *Horizon 2020 project that studied research integrity from an organisational and institutional perspective, among others leading to an online training course UPRIGHT.*
- [*VIRT2UE*](https://www.embassy.science/training) *Horizon 2020 project that* is developing a new blended learning train the trainer programme on Ethics and Research Integrity (ERI) to form new trainers and give them tools to internalize, apply and uphold the principles of the European Code of conduct for Research Integrity

### Evaluation

- [Declaration on Research Assessment (DORA)](https://sfdora.org) *Global guidelines for improving the assessment of research and evaluation of researchers*

- [Leiden Manifesto for Research Metrics](http://www.leidenmanifesto.org) *10 principles to improve the measurement of research performance from Leiden University*

### Rewards

- [Open science badges](https://cos.io/our-services/open-science-badges/)
  *Effectively, these are small icons printed at the first page of an article to indicate if an article contains open data, materials, and/or is preregistered.*
- [The Reward Alliance](http://rewardalliance.net/)
  *Specifically, Cochrane has created the* [*Cochrane-REWARD prize*](http://rewardalliance.net/2018-cochrane-reward-prize/) *that highlights both underused "remedies" against research waste and the need to invest in research to identify problems and solutions to them.*
- New reward structures
  *University of Ghent has recently implemented* [*new reward structures*](https://www.nwo.nl/algemeen/actueel/social-media/onderzoek-online/2019-1-anders-waarderen) *for its employees, now rewarding a more diverse set of academic practices, partly decided on by the researchers themselves.*

### Funding

- [NWO Replication Grant](http://www.nwo.nl/replicationstudies)
  *The Dutch funding agency NWO started a grant specifically meant to fund replication research*
- [Inclusive funding](https://www.nwo.nl/actueel/nieuws/2018/11/vernieuwing-in-het-waarderen-en-belonen-van-wetenschappers.html)
  NWO*, VSNU, NFU and ZonMw are currently investigating how novel funding structures can be more inclusive. They aim to set up funding scheme’s that enables more diverse career tracks, innovates the evaluation mechanisms and fosters team science.*

## The Empirical Cycle

### Meta-analysis

- Publication bias detection/correction methods
  - [P-curve](http://pcurve.com)
  - [P-uniform](https://rvanaert.shinyapps.io/p-uniform/)

### Replication

- [NWO Replication Grant](http://www.nwo.nl/replicationstudies)
  *The Dutch funding agency NWO started a grant specifically meant to fund replication research.*
- [Collaborative Replications and Education Project (CREP)](https://osf.io/wfc6u/)
  *This is a replication project where students are encouraged to conduct replications as part of their courses.*
- Replications through multi-lab collaborations
  - [Reproducibility Project: Psychology](https://science.sciencemag.org/content/349/6251/aac4716)
    *Collaborative effort to document the replicability of studies in psychology.*
  - [Reproducibility Project: Cancer Biology](https://www.ncbi.nlm.nih.gov/pmc/articles/PMC4270077/)
    *Collaborative effort to document the replicability of studies in cancer biology.*
  - Many Labs

*A series of collaborative efforts to replicate sets of psychological experiments. See e.g.,* [*Many Labs 2*](https://osf.io/8cd4r/)*.*

- - [Registered Replication Reports](https://www.psychologicalscience.org/publications/replication)
    *Pregistered, multi-lab replication studies. This is a subset of the Registered Reports format.*
  - [Psychological Science Accelerator](https://psysciacc.org/)
    *A globally distributed network of psychological science laboratories that coordinates data collection for democratically selected studies.*
- The “[Pottery Barn Rule](https://blogs.royalsociety.org/publishing/reproducibility-meets-accountability/)” in the journal *Royal Society Open Science
  This is the guarantee to publish any close replication of any study previously published in the same journal.*
- KNAW report “[Improving Reproducibility in the Empirical Sciences](https://www.knaw.nl/en/news/publications/replication-studies)”
  *Report analyzing causes for non-replication and offering recommendations for improving reproducibility and conducting replication studies.*

### Peer review

- [RetractionWatch](http://retractionwatch.com/)
  *Popular blog tracking scientific retractions and covering cases of misconduct.*
- [PubPeer](https://pubpeer.com/static/about)
  *Online platform for post-publication peer review.*
- [Registered Reports](https://cos.io/rr/)
  *Publication format where peer review takes place before data are collected and articles are accepted regardless of the results.*
- [Exploratory Reports](https://www.sciencedirect.com/science/article/pii/S0010945217302393)
  *Publication format specifically meant for exploratory research (as opposed to confirmatory, hypothesis-testing research)*
- Open peer review
  *Peer reviews are published alongside the article. See e.g., the journal* [*Royal Society Open Science*](https://royalsocietypublishing.org/rsos/for-reviewers#question7)*.*
- [Peer Reviewers’ Openness (PRO) Initiative](https://opennessinitiative.org/)
  *Signatories of this initiative only accept review invitations if the manuscript adheres to open practices or states why it does not.*
- [Peer Community](https://peercommunityin.org/)
  *A platform for peer reviewing and publishing preprints.*

### Publication systems

- [F1000 research](https://f1000research.com/)
  *F1000Research is an Open Research publishing platform for life scientists*
- [Publons](https://publons.com/about/home/) *Platform that allows researchers to track publications, citation metrics, peer reviews and journal editing work.*
- [Journal of Open Psychology Data](https://openpsychologydata.metajnl.com/)
  *The Journal of Open Psychology Data (JOPD) features peer reviewed data papers describing psychology datasets with high reuse potential.*

### Preprints

- Preprint servers
  *Online archives to publish manuscript versions that have not undergone peer review yet. Preprint servers are also often used to publish post-prints: the non-edited version of a published paper. Examples are:*
  - [*ArXiv*](https://arxiv.org)
  - [*PsyArXiv*](https://psyarxiv.com)
  - [*BioArXiv*](https://www.biorxiv.org)
  - *For a more complete list, see* [*https://osf.io/preprints/*](https://osf.io/preprints/)
- [Peer Community](https://peercommunityin.org/)
  *A platform for peer reviewing and publishing preprints.*

### Open Access

- [Plan S](https://www.coalition-s.org)
  Initiative by a coalition of research funders and charities to open up research publications
- [Dutch Open Access Deals](https://www.openaccess.nl/en/in-the-netherlands/publisher-deals)
  Agreements between Dutch university libraries and scholarly publishers for Open Access

### Preregistration

- [AsPredicted](https://aspredicted.org)
  *A website to generate and publish standardized preregistrations*
- [Open Science Framework](https://osf.io)
  *A platform that allows sharing all steps and products of the research process and publicly preregistering research plans.*
- [Registered Reports](https://cos.io/rr/)
  *Publication format where peer review takes place before data are collected and articles are accepted regardless of the results.*
- [Registered Replication Reports](https://www.psychologicalscience.org/publications/replication)
  *Pregistered, multi-lab replication studies. This is a subset of the Registered Reports format.*

### Multi-lab collaborations

- [Reproducibility Project: Psychology](https://science.sciencemag.org/content/349/6251/aac4716)
  *Collaborative effort to document the replicability of studies in psychology.*
- [Reproducibility Project: Cancer Biology](https://www.ncbi.nlm.nih.gov/pmc/articles/PMC4270077/)
  *Collaborative effort to document the replicability of studies in cancer biology.*
- Many Labs
- *A series of collaborative efforts to replicate sets of psychological experiments. See e.g.,* [*Many Labs 2*](https://osf.io/8cd4r/)*.*
- [Study Swap](https://osf.io/view/studyswap/)
  *Platform to exchange resources (such as lab-time) between labs across the world.*
- [Registered Replication Reports](https://www.psychologicalscience.org/publications/replication)
  *Pregistered, multi-lab replication studies. This is a subset of the Registered Reports format.*
- [Psychological Science Accelerator](https://psysciacc.org/)
  *A globally distributed network of psychological science laboratories that coordinates data collection for democratically selected studies.*

### Data sharing

- [Journal of Open Psychology Data](https://openpsychologydata.metajnl.com/)
  *The Journal of Open Psychology Data (JOPD) features peer reviewed data papers describing psychology datasets with high reuse potential.*
- Data management; DMPonline
- [Open Science Framework](https://osf.io)
  *A platform that allows sharing all steps and products of the research process and publicly preregistering research plans.*
- [GO Fair](https://www.go-fair.org)
  *Initiative that aims to implement the FAIR principles: making data Findable, Accessible, Interoperable, and Reusable*
- [figshare](about:blank)
  *figshare helps academic institutions store, share and manage all of their research outputs*
- [The Dataverse Project](https://dataverse.org/)
  *Open source research data repository software*
- [Transparency and Openness Promotion (TOP) guidelines](https://cos.io/top/)
  *Eight transparency standards of which journals can indicate a level of implementation*
- Open Sciences badges

In remains uncertain if this approach works and is effective ([link](https://doi.org/10.1371/journal.pbio.1002456PMID:2717100749) & [link](https://doi.org/10.31234/osf.io/d8wex)). Research is inconclusive, but interesting to follow *up.*

### Reproducible workflows

- [Open Science Framework](https://osf.io)
  *A platform that allows sharing all steps and products of the research process and publicly preregistering research plans.*
- [Jupyter Notebook](https://jupyter.org/)
  *“The Jupyter Notebook is an open-source web application that allows you to create and share documents that contain live code, equations, visualizations and narrative text.”*
- [R Markdown](https://rmarkdown.rstudio.com/)
  *R Markdown is a file format that allows the user to create dynamic documents in which narrative text and code are interweaved. This format greatly increases (analytical) reproducibility.*
- [Code Ocean](about:blank)
  *Code Ocean is an online platform where users can develop and share code through a web browser in a fully reproducible environment.*
- Journals who check computational reproducibility of submitted papers
  *For a list, see* <https://osf.io/kgnva/wiki/home/>
- Journal reporting guidelines
  - [CONSORT](https://apastyle.apa.org/manual/related/moher-2001.pdf)
    *Reporting guidelines for randomized controlled trials*

- - [PRISMA](http://www.prisma-statement.org/), [MARS](https://wmich.edu/sites/default/files/attachments/u58/2015/MARS.pdf)
    *Reporting guidelines for meta-analyses and systematic reviews*
  - [*EQUATOR Network*](https://www.equator-network.org)

*Library of reporting guidance*

### Statistical innovations

- [JASP](https://jasp-stats.org); [jamovi](https://www.jamovi.org/)
  *Free, statistical software that allows for both frequentist and Bayesian analysis*
- [Statcheck](http://statcheck.io), [GRIM](https://peerj.com/preprints/2064/), [GRIMMER](https://peerj.com/preprints/2400/), [GRIMMEST](http://www.omnesres.com/research/grimmest/), [SPRITE](https://peerj.com/preprints/26968/)
  *Free software to check for statistical inconsistencies in papers, without needing access to raw data*
- Journal guidelines on improved statistical inference

*E.g.,* [*the new statistical guidelines for journals of the Psychonomic Society*](https://www.springer.com/psychology?SGWID=0-10126-6-1390050-0)

*- METRICS Institute (*[*link*](https://metrics.stanford.edu/)*)*

- Free online statistics courses (MOOCs)
  *E.g., the Coursera course “*[*Improving your statistical inference*](https://www.coursera.org/learn/statistical-inferences)*”*
- [Myth of NHST](https://www.themythofnhst.com/)
  *Dutch research project investigating researchers’ views on the use of NHST or alternative methods and what they see as the advantages and disadvantages of these methods.*
- Publication bias detection/correction methods
  - [P-curve](http://pcurve.com)
  - [P-uniform](https://rvanaert.shinyapps.io/p-uniform/)

### Retractions

- [RetractionWatch](http://retractionwatch.com/)
  *Popular blog tracking scientific retractions and covering cases of misconduct.*

# Appendix III: Full list of ranked themes that we have indicated as gaps of knowledge by the authors of the position paper (n=4)

| **List of themes that are currently underrepresented in our topic list (in ranked order n=4 authors):** |
| --- |
| 1. Responsible assessment of researchers 2. Research on responsible mentoring, supervision and role modeling 3. The influence of Open Science/Transparency 4. Effect of education/training of Responsible Research Practices 5. Responsible and fair peer Review 6. The influence and implementation of preregistrations 7. Checking of reproducibility 8. Responsible funding 9. Monitoring of the research process 10. Consequences of power and hierarchy structures 11. Publication bias 12. Responsible reporting of research 13. Consequences of research misconduct 14. Statistical inference and analysis 15. Credit and authorship issues 16. Performing replication studies 17. The influence of diversity-issues on responsible research 18. The use of theories for methodology |

# Appendix IV: Biographies

Serge Horbach
Serge Horbach works as a postdoc at the Institute for Science in Society, Radboud University, and the Centre for Science and Technology Studies, Leiden University. His research focuses on research integrity and the scientific publication system, working in a sociology of science and science and technology studies tradition. Currently he studies how erroneous or fraudulent research may enter the scientific literature and how editorial and peer review practices may be organised to prevent this. Wider research interests include scientific (e)valuation practices and the impact of misidentified biospecimen on replicability.

Michèle Nuijten
Michèle Nuijten is an Assistant Professor in Methodology and Statistics at Tilburg University, where she is part of the Meta-Research Center. Michèle obtained her Bachelor’s (2011) and Master’s (2012) in Psychological Methods at the University of Amsterdam. In her PhD thesis (2018, Tilburg University), she focused on meta-scientific studies of problems and solutions in psychological science. Among other things, she co-developed the tool “statcheck”: a spellchecker for statistics. Currently, her work focuses on reproducibility and replicability in psychology.

Gareth O’Neill
Gareth O’Neill is a doctoral candidate in linguistics at Leiden University and has represented early-career researchers extensively in the Netherlands and in Europe. He is former president of the European Council of Doctoral Candidates and Junior Researchers (Eurodoc) and has been active as contributor to the Dutch National Plan for Open Science (NPOS), expert on Open Science for the European Commission, and advisory board member for the FOSTER Plus project. He is currently an ambassador for Plan S for cOALition S and adviser for the European Open Science Cloud (EOSC).

Joeri Tijdink
Joeri Tijdink is an Assistant Professor at the metamedica department at Amsterdam UMC, location VUmc and philosophy department at VU University. He obtained his PhD in 2016 with the thesis entitled Publish & Perish; research on research and researchers. Currently he is involved in several research projects that investigate research integrity, publication pressure, research culture and responsible research practices and is the author of the self help guide: Scientist on the Sofa; how to survive at the university. He also works as a clinical psychiatrist.
